# Visible Light Motivated the Photocatalytic Degradation of P-Nitrophenol by Ca^2+^-Doped AgInS_2_

**DOI:** 10.3390/molecules29020361

**Published:** 2024-01-11

**Authors:** Xuejiao Wang, Shuyuan Liu, Shu Lin, Kezhen Qi, Ya Yan, Yuhua Ma

**Affiliations:** 1 College of Pharmacy, Dali University, Dali 671000, China; 15187277683@163.com (X.W.); liushuyuan@symc.edu.cn (S.L.); lin_s@outlook.com (S.L.); 2 College of Chemistry and Chemical Engineering, Xinjiang Normal University, Urumqi 830054, China

**Keywords:** AgInS_2_, Ca^2+^, doping, 4-Nitrophenol, visible photocatalytic degradation

## Abstract

4-Nitrophenol (4-NP) is considered a priority organic pollutant with high toxicity. Many authors have been committed to developing efficient, green, and environmentally friendly technological processes to treat wastewater containing 4-NP. Here, we investigated how the addition of Ca^2+^ affects the catalytic degradation of 4-NP with AgInS_2_ when exposed to light. We synthesized AgInS_2_ (AIS) and Ca^2+^-doped AgInS_2_ (Ca-AIS) with varying amounts of Ca^2+^ using a low-temperature liquid phase method. The SEM, XRD, XPS, HRTEM, BET, PL, and UV-Vis DRS characteristics were employed to analyze the structure, morphology, and optical properties of the materials. The effects of different amounts of Ca^2+^ on the photocatalytic degradation of 4-NP were investigated. Under visible light illumination for a duration of 120 min, a degradation rate of 63.2% for 4-Nitrophenol (4-NP) was achieved. The results showed that doping with an appropriate amount of Ca^2+^ could improve the visible light catalytic activity of AIS. This work provides an idea for finding suitable cheap alkaline earth metal doping agents to replace precious metals for the improvement of photocatalytic activities.

## 1. Introduction

Environmental pollution has been intensified recently due to the increasing global population and frequent human activities. While people enjoy the benefits of chemical products and natural mineral resources, they have enforced the threat of toxic organic pollutants. Huge amounts of chemical wastes are discharged into the environment every year and cause great harm to the environment [1]. 4-Nitrophenol (4-NP) is a frequently used herbicide and fungicide that is widely used in medicine, dyes, and agricultural activities [2]. It is a highly toxic and insoluble organic pollutant in the environment, which has been detected in surface water and soil and even in beverages and food. It causes great harm to the human body and environment [3,4]. 4-NP is a member of 120 blacklisted priority pollutants by the U.S. Environmental Protection Agency. The delocalization of π electrons in the benzene ring makes 4-NP highly stable [5]. Therefore, finding a safe and efficient approach to degrade 4-NP is of utmost importance.

Common methods for the removal of 4-NP include photocatalytic oxidation [6], adsorption, and biological methods [7,8]. However, most of these technologies require further separation and purification steps to process substantial quantities of residual waste, potentially resulting in secondary pollution [9]. Photocatalytic oxidation has been proven to effectively degrade 4-NP into CO_2_, H_2_O, or other non-toxic compounds, with mild reaction conditions, low cost, and high efficiency [10,11]. During photocatalysis, the irradiation of the semiconductor produces excited electrons and holes, respectively, in the conduction and valance bands of the photocatalyst. These excited charges directly oxidize and reduce the target pollutant on the surface of the photocatalyst with the aid of highly reactive degrading species [12]. Amongst the various photocatalysts, TiO_2_ is a widely used catalyst with many attractive characteristics. However, the band gap of TiO_2_ is wide (3.23 eV), limiting its ability to catalyze redox reactions under visible light irradiation [13]. By modifying TiO_2_ or seeking new visible light-driven catalysts, there are several methods available for improving the catalytic performance of a catalyst: doping with metal or non-metallic ions, adjusting the morphology, loading precious metals, and utilizing composite semiconductors. Each of these approaches has been proven to enhance the efficiency and effectiveness of catalysts in various applications. By understanding and utilizing these methods, researchers can develop catalysts with improved performance for a wide range of chemical reactions [14,15].

As the I-III-VI ternary direct band gap semiconductor, AgInS_2_ has attracted extensive attention due to its excellent light absorption characteristics, appropriate band gap width, high absorption coefficient, good radiation stability, and nonlinear optical properties [16]. There are two different polymorphs in AgInS_2_ crystals: a tetragonal chalcopyrite structure at room temperature and an orthorhombic wurtzite structure at high temperature [17]. Furthermore, it is a non-toxic and environmentally friendly visible light sensitizer and has been used in fluorescence, solar cells, and photocatalysis, and it has garnered significant attention [18,19,20]. AgInS_2_ has three phase structures: cubic, tetragonal, and orthogonal. Its wurtzite structure is composed of InS_4_ and AgS_4_ tetrahedrons in the orthorhombic system [21]. The unequal bonds between Ag-S and In-S can cause the tetrahedrons to twist, resulting in an internal electric field [22]. Under illumination, the electric field is conducive to the separation of charges and enhances photocatalytic activity. Its optical band gap (1.80~2.04 eV) is close to the optimal forbidden bandwidth (1.45 eV) of solar cell materials, making it an ideal choice as a photocatalytic material [23]. Although AgInS_2_ has many advantages, its high charge recombination rate, low quantum efficiency, and strong photoetching badly limit its widespread application in the photocatalytic field. Therefore, it is essential to modify the AgInS_2_ monomer and expand its application in the photocatalytic field. As a common cheap alkaline earth metal, calcium is used as a dopant in many semiconductors to reduce the band gap or interface resistance [24,25,26]. Based on the above analysis, it was speculated that AIS has certain degradation activity toward 4-NP under visible light. After introducing Ca^2+^, the band gap and interface resistance of AIS could be reduced, thereby improving the photocatalytic activity of AIS. Given that the radius of Ca^2+^ (99 pm) is smaller than Ag^+^ (115 pm) and larger than In^3+^ (80 pm) [27,28,29], it is speculated that after the introduction of Ca^2+^, it replaces Ag^+^ in entering the lattice of AIS. This modulation of the lattice is believed to adjust the band gap of AIS, thereby enhancing its degradation activity toward 4-NP under visible light. 

The present study aims to explore the effect of introducing Ca^2+^ on the degradation of 4-NP by AIS under visible light, and the low-temperature liquid phase method was selected to synthesize AIS and Ca-AIS in different proportions. The influences of Ca doping on its morphology, band gap, and the electron hole recombination of AIS were also analyzed. The findings demonstrated that the presence of an appropriate amount of Ca^2+^ enhances the photocatalytic degradation performance of AIS. Herein, the potential mechanism for the photocatalytic degradation of 4-NP using Ca-doped AIS was proposed. We believe that this work will substitute precious metals as dopants to improve the performance of photocatalysts. Compared to noble metal elements, Ca is cheaper and can modify photocatalysts with less doping to improve its photocatalytic activity. Moreover, this method is easy to operate and requires lower cost.

## 2. Results and Discussion

### 2.1. Crystal Structure

**Figure 1:**
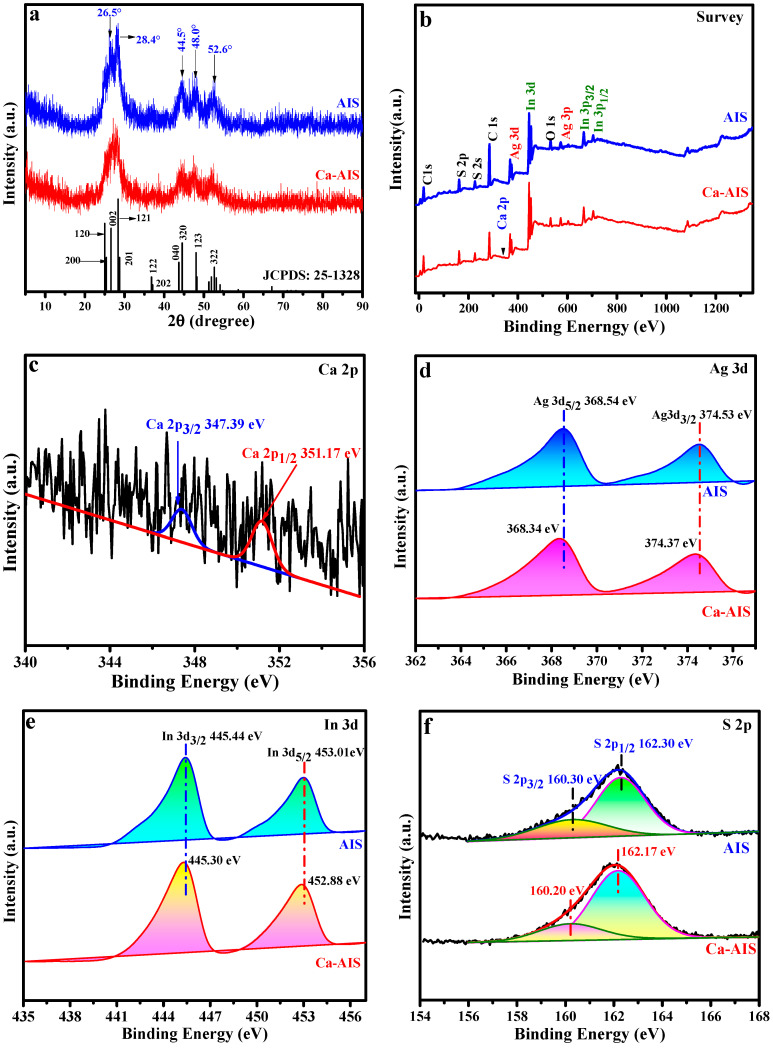
(**a**) XRD patterns of AIS and 1%Ca-AIS, (**b**) survey XPS spectrum and high-resolution XPS spectrum of AIS and 1%Ca-AIS: (**c**) Ca 2p; (**d**) Ag 3d; (**e**) In 3d; (**f**) S 2p.

To examine the crystal characteristics and phases of the samples, XRD analysis was conducted with AIS and 1%Ca-AIS samples. According to Figure 1a, the pure AIS sample exhibits distinct diffraction peaks at 26.5°, 28.4°, 44.5°, 48.0°, and 52.6°. These peaks correspond to crystal planes (002), (121), (320), (123), and (322), respectively, as indicated in the standard AIS spectrum with an orthogonal crystal structure (JCPD: 25-1328) [30]. Interestingly, no peaks related to impurities are detected, implying the successful synthesis of AIS. Upon the introduction of Ca^2+^, there is no observable Ca-related peak in the XRD spectrum of the 1%Ca-AIS sample. The absence could be attributed to the low concentration of the Ca^2+^ dopant. Notably, the positions of all diffraction peaks remain unchanged, suggesting that Ca^2+^ does not significantly affect the crystal structure of AIS. However, the intensity of the characteristic diffraction peaks is slightly weakened, indicating that the introduction of Ca^2+^ can influence the crystallinity of AIS. These findings possibly suggest that Ca^2+^ is evenly distributed within the AIS matrix. Furthermore, the introduction of Ca^2+^ leads to the broadening of the characteristic diffraction peaks. This phenomenon is likely due to the smaller radius of Ca^2+^ (99 Å) compared to Ag^+^ (115 Å) [28,31]. Based on the analysis mentioned above, it can be inferred that Ca^2+^ replaces Ag^+^ within the AIS lattice upon introduction. Consequently, this substitution results in a reduction in the grain size of AIS [32].

The chemical composition and surface chemical state of AIS and 1%Ca-AIS samples were examined using XPS. As shown in Figure 1b, AIS contains not only Ag, In, and S but also O, C, and other elements. The C and O elements are produced in thioglycolic acid, the coating agent of the material, or the air. The obtained samples have no impurities, and the peak positions of Ag, In, and S are consistent with the previous report [33]. Figure 1c–f presents the high-resolution spectra of different elements in 1%Ca-AIS. Figure 1c reveals that the concentration of the doped Ca element is quite low, and a minimal amount of Ca is observed on the surface. The two main peaks of Ca 2p can be fitted, and a binding energy peak at 347.39 eV is ascribed to Ca 2p_3/2_, while another binding energy peak at 351.17 eV is associated with Ca 2p_1/2_. These findings indicate that Ca is present in the form of Ca^2+^ [34,35,36]. In Figure 1d, the two binding energy peaks at 374.53 eV and 368.54 eV are attributed to Ag 3d_3/2_ and Ag 3d_5/2_, respectively. This implies that the Ag in AIS has a valence state of +1 [37]. The Ca-AIS sample reveals distinguishable binding energy peaks of Ag 3d_3/2_ (374.37 eV) and Ag 3d_5/2_ (368.34 eV). The observed results propose that the presence of Ca^2+^ ions leads to a decrease in the binding capacity of Ag^+^ ions toward the electrons existing in the crystal structure. The high-resolution spectrum of In 3d in Figure 1e exhibits two binding energy peaks at 445.44 eV and 453.01 eV, which correspond to In 3d_3/2_ and In 3d_5/2_, respectively [38]. After the incorporation of Ca^2+^, a shift in the binding energy peaks can be observed, where the binding energy values for In 3d_3/2_ and In 3d_5/2_ undergo a change to 445.30 eV and 452.88 eV, respectively. This suggests that the presence of Ca reduces the binding strength of In^3+^ within the crystal lattice. These results demonstrate that Ca^2+^ replaces Ag^+^ and enters the AIS lattice, resulting in the weakening of the binding ability of Ag^+^ and In^3+^ to electrons. In Figure 1f, the peak corresponding to the binding energy of S 2p_3/2_ is detected at approximately 160.30 eV, while the peak for S 2p_1/2_ is observed at 162.30 eV. These results imply that the valence state of S in AIS is −2 [39,40,41]. Upon the introduction of Ca^2+^, the binding energy peak of S 2p_3/2_ shifts to around 160.20 eV, and the binding energy of S 2p_1/2_ moves to 162.17 eV. This suggests a reduction in the binding affinity of S toward electrons after the introduction of Ca^2+^. The XPS characterization results of the samples confirm that Ca^2+^ replaces Ag^+^ in AIS. Although the oxidation states of the elements in AIS are unaffected by the introduction of Ca^2+^, the binding ability of Ag, S, and In to electrons is reduced.

### 2.2. Morphology and Structure

**Figure 2:**
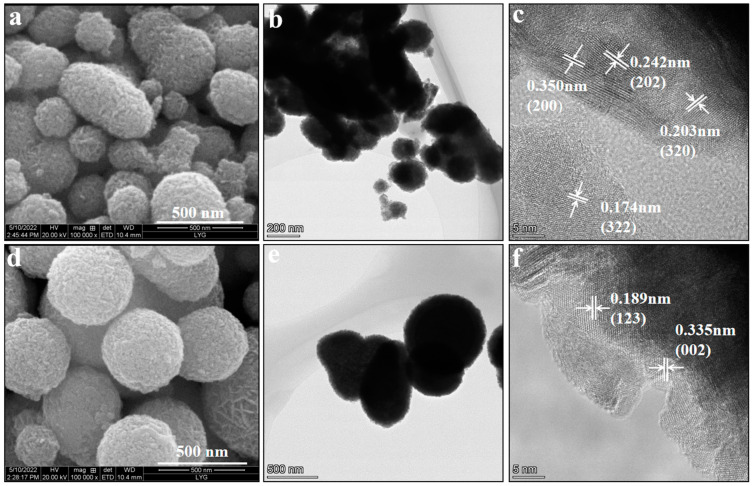
SEM, TEM, and HRTEM images of AIS (**a**–**c**) and 1%Ca-AIS (**d**–**f**).

SEM was used to examine the microscopic morphology of 1%Ca-AIS and AIS. Figure 2a indicates that the prepared AIS particles have rough surfaces, different shapes, uneven size, and serious agglomerations. AIS exhibits a particle size ranging from 361 to 630 nm. In Figure 2d, it is apparent that 1%Ca-AIS particles present smoother surfaces, improved sphericity, and a more uniform dispersion among the particles. The particle size distribution of Ca-AIS exhibits uniformity, with the average diameter ranging between 253 and 481 nm. According to SEM analysis, the doping of Ca^2+^ makes the surface of AIS smooth, the sphericity becomes better, the particle size becomes smaller, the size is more uniform, and the dispersion is better.

To further investigate the morphology and crystal lattice arrangement of both AIS and 1%Ca-AIS samples, a high-resolution TEM (HRTEM) analysis was conducted. The HRTEM analysis showcased in Figure 2b portrays the morphology of AIS, illustrating an aggregate structure with irregular particle sizes ranging from approximately 137 to 222 nm. As shown in Figure 2c, the measured interplanar spacings are 0.350 nm, 0.242 nm, 0.203 nm, and 0.174 nm, which corresponds to (200), (202), (320), and (322) crystal planes of AIS with an orthogonal crystal phase structure, respectively. According to the data displayed in Figure 2e, the addition of Ca^2+^ has a distinct impact on the characteristics of Ca-AIS particles. The Ca-AIS particles exhibit a smooth surface and possess a favorable sphericity, with an average size ranging from 106 to 166 nm. Figure 2f showcases clear lattice patterns on the surface of the particles, indicating the presence of well-defined crystal structures. The measured interplanar spacings of 0.189 nm and 0.335 nm that correspond to (123) and (002) crystal planes, respectively, are attributed to the orthogonal crystal phase of AIS. These findings are consistent with the obtained XRD results, affirming the accuracy and reliability of the HRTEM data.

### 2.3. BET

**Figure 3:**
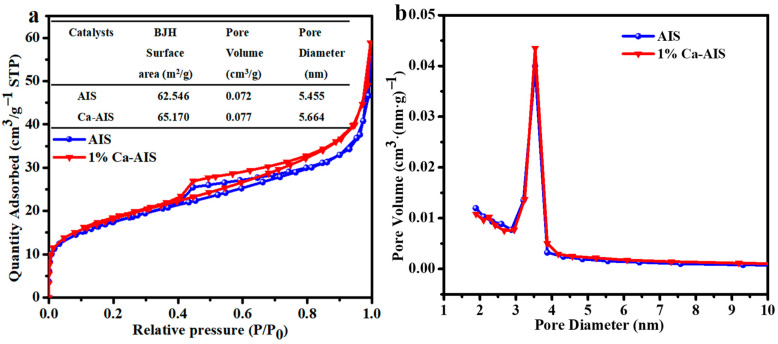
(**a**) N_2_ adsorption–desorption isotherms and (**b**) corresponding pore size distributions of AIS and 1%Ca-AIS.

Nitrogen adsorption–desorption tests were employed to analyze the pore size and specific surface area of AIS and 1%Ca-AIS. The isotherms depicted in Figure 3a exhibit typical type IV characteristics with H3 hysteresis loops in the P/P_o_ range of 0.4 to 1.0. These findings imply that both AIS and 1%Ca-AIS possess mesoporous structures [26,42]. The specific surface area of 1%Ca-AIS is slightly larger than AIS. A higher specific surface area is known to enhance photocatalytic activity and increase the number of active sites. Consequently, this suggests that 1%Ca-AIS may exhibit improved photocatalytic performance compared to AIS. Additionally, Figure 3b reveals that the introduction of Ca^2+^ increases the pore size, which, in turn, increases the effective contact area with pollutants and effectively enhances the adsorption performance of AIS.

### 2.4. Electronic Structure

**Figure 4:**
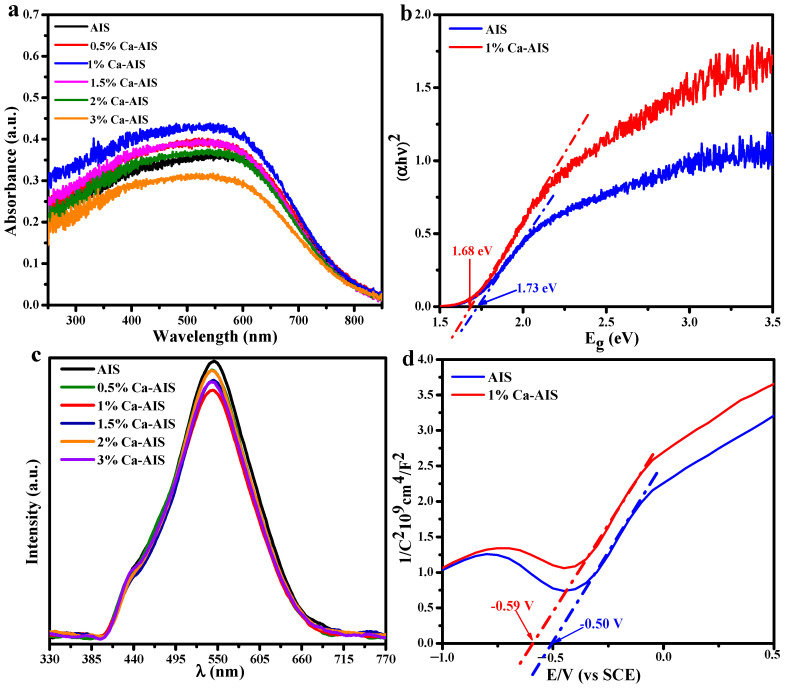
(**a**) UV–Vis DRS spectra, (**b**) band gap energy diagram, (**c**) fluorescence spectrum, (**d**) flat band potential diagrams of AIS and Ca-AIS.

To evaluate the capacity of the samples to absorb visible light, UV-vis DRS characterizations of the samples were conducted. As depicted in Figure 4a, it is evident that Ca-AIS proportions demonstrate significant light absorption within the 400–700 nm range. Except for 3%Ca-AIS, which shows lower light absorption capacity compared to AIS, the other proportions of Ca-AIS demonstrate higher light absorption capacity than AIS, indicating that the introduction of the appropriate amount of Ca^2+^ is indeed conducive to the absorption of visible light. However, excessive Ca^2+^ will inhibit its optical utilization. The bandgap widths of AIS and 1%Ca-AIS were calculated according to Tauc’s function by the Kubelka–Munk (KM) method: αhν = A(hν − Eg)^n/2^, where α is the absorbance coefficient of the substance, h is the Planck constant, A is the absorbance of the sample, Eg is the forbidden bandgap width of the substance, ν is the photon frequency [43,44], and n is equal to 1 or 4, and its value depends on the direct or indirect type conversion of the semiconductor. As we all know, AgInS_2_ belongs to the direct transition semiconductor, and the value of n is 1 [45]. The relationship between (αhν)^2^ and hν is illustrated in Figure 4b. By estimating and calculating the intercept of the tangent line with the curve, the band gap value (E_g_) was determined as the point where the tangent intersects the x-axis, and it is obtained that the band gap energy (~1.68 eV) of 1%Ca-AIS is lower than AIS (~1.73 eV). Therefore, the enhanced photocatalytic efficiency of 1%Ca-AIS may be attributed to its strong light absorption.

The photoluminescence spectrum provides information about the separation effect of photogenerated electrons and holes. Figure 4c illustrates the fluorescence spectra of all samples, with the following measurement parameters: an excitation wavelength of 400 nm and a slit width of EX: 6HH and EM: 10. The results demonstrate that AgInS_2_ exhibits the strongest emission peak at 544 nm. Upon the introduction of Ca^2+^, the fluorescence intensity is weakened, as Ca^2+^ inhibits the recombination of e^−^ and h^+^. Notably, the fluorescence intensity of 1%Ca-AIS is the weakest, indicating better separation of electrons and holes, and the lifetime of photogenerated carriers is the longest, which indicates that the introduction of Ca^2+^ reduces the recombination rate of carriers facilitating the separation of photogenerated electrons and holes. This is beneficial for the photocatalytic degradation of pollutants. The PL spectrum further confirms that the separation rate of electron-hole pairs aligns with the corresponding efficiency of degradation, highlighting the enhanced ability of AIS to degrade organic pollutants upon the introduction of Ca^2+^.

The impedance potential approach was employed to evaluate the flat band potential of the specimens. Specifically, AIS catalysts and 1%Ca-AIS catalysts were subjected to a series of experiments to determine their respective flat band potentials. The acquired data were reversed employing the Mott–Schottky theory, yielding Mott–Schottky curves for both AIS and 1%Ca-AIS catalysts. As depicted in Figure 4d, the patterns exhibit positive slopes, indicating that the specimens exhibit characteristics of n-type semiconductors. The point of intersection between the tangent lines of the Mott–Schottky curves and the x-axis yield values of −0.50 V and −0.59 V for AIS and 1%Ca-AIS catalysts, respectively, utilizing Formula (2) [29]:

(1) $$E_{0}=E_{fb}+\frac{RT}{F}$$

The point of intersection between the tangent of the Mott–Schottky curve and the x-axis is called intercept E_0_. R represents the standard molar gas constant, the thermodynamic temperature is denoted as T, and the Faraday constant is represented by F. The flat band potentials (E_fb_) for AIS and 1%Ca-AIS are determined as −0.53 V and −0.62 V (vs. SCE), respectively. The conduction band potentials of AIS and 1%Ca-AIS can be determined using a specific Formula (3):

(2) $$E_{CB}=E_{\mathrm{fb}}-kT\ln\frac{Nc}{N}$$

In this context, k denotes the Boltzmann constant, Nc indicates the effective state density of the conduction band, and N represents the doping concentration. Through calculations, the value of kTln(Nc/N) is approximately 0.1 eV. Consequently, the conduction band potentials (E_CB_) for AIS and 1%Ca-AIS are found to be −0.63 V and −0.72 V, respectively. Furthermore, utilizing empirical Formula (4), the valence band potential (E_VB_) of the semiconductor was calculated as follows [46]:

E_CB_ = E_VB_ − E_g_ (3)

In this formula, E_CB_ represents the conduction band potential. The band gap energy is represented by Eg, and it determines the valence band potentials of AIS and 1%Ca-AIS. The calculated valence band potential values are 1.10 V for AIS and 0.96 V for 1%Ca-AIS.

### 2.5. Photoelectric Property and Photocatalytic Activity

**Figure 5:**
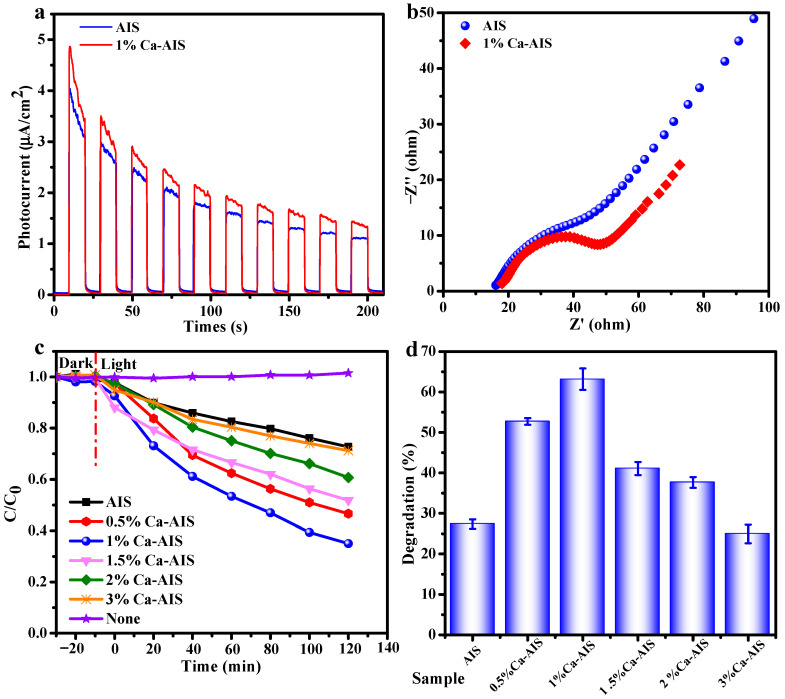
(**a**) Photocurrent response, (**b**) electrochemical impedance spectrum of AIS and 1%Ca-AIS, (**c**) photocatalytic removal curves, and (**d**) degradation rates of 4-NP by AIS and Ca-AIS under visible light irradiation over 120 min.

The transient photocurrent response (I-t) curves and electrochemical impedance spectroscopy (EIS) provide a comprehensive understanding of the separation of electrons and holes in the sample. By analyzing the movement and behavior of charge carriers, we can identify limitations and opportunities for improvement in various applications, such as solar cells and photocatalysis. Figure 5a shows the diagrams of transient photocurrent response for AIS and 1%Ca-AIS. When the lamp is turned on for the first time, both AIS and 1%Ca-AIS exhibit good photocurrent response in the test, and the photocurrent intensity of 1%Ca-AIS (~2.920 μA/cm^2^) is stronger than AIS (~2.56 μA/cm^2^). Upon turning off the light, the current intensity of AIS and 1%Ca-AIS decreases rapidly. After performing the switch on and off for 10 consecutive operations, it becomes evident that both AIS and 1%Ca-AIS maintain excellent reproducibility in terms of photocurrent response. This observation implies the swift segregation of electrons and holes within the material. However, the photocurrent response of AIS and 1%Ca-AIS gradually decreases, which may be due to the partial loss of electrons in the closed circuit under current excitation. Nevertheless, the response intensity of 1%Ca-AIS remains higher than AIS, suggesting a better separation efficiency of electrons and holes in 1%Ca-AIS. This implies that there are more electrons in 1%Ca-AIS to participate in the catalytic reaction process, which proves that the introduction of Ca^2+^ can enhance the photocatalytic effect.

An examination of electrochemical impedance through the utilization of the “AC impedance” approach was conducted. The open circuit potential served as the primary voltage, maintaining stability throughout the testing process. For this particular experiment, a solution of 0.1 mol/L KCl incorporating 5 mm K_3_[Fe(CN)_6_] and K_4_[Fe(CN)_6_] was selected as the electrolyte solution. Figure 5b shows the EIS Nyquist curve obtained from this test. The charge transfer process occurring at the interface between the electrode and the electrolyte can be depicted by the arc observed in the EIS Nyquist curve. Typically, a decrease in the radius of the arc indicates a lower transfer resistance for carriers, thereby leading to enhanced separation and transmission efficiency of photogenerated electrons and holes [43]. The EIS Nyquist diagram reveals that the arc radius of 1%Ca-AIS is smaller compared to AIS. This signifies that the addition of Ca^2+^ improves electronic conductivity. It achieves this by efficiently segregating photogenerated carriers and promoting the swift transfer of interface charges to enhance the overall conductivity. Consequently, 1%Ca-AIS exhibits higher electronic conductivity and realizes the rapid transfer of interface charges, thus showing better photocatalytic activity.

Figure 5c depicts dark adsorption and photocatalytic degradation curves for both Ca-AIS and AIS under visible light. In the absence of light, the concentration of the 4-NP solution remains relatively constant with minimal changes. In Figure 5d, it is evident that the degradation rate of 4-NP under visible light without Ca^2+^ doping is 27.4% for AIS. However, upon the introduction of Ca^2+^, the degradation rates of 4-NP under visible light for Ca-AIS increase significantly, with doping contents of 0.5% (52.7%), 1% (63.2%), 1.5% (41.1%), and 2% (37.6%). All these degradation rates are higher than pure AIS. Remarkably, the degradation rate of 4-NP with a doping content of 3% is slightly lower than AIS, measuring at 24.9%. Importantly, all the samples exhibit remarkable reproducibility in photocatalytic degradation. These experimental findings support the notion that pure AIS has the capability to harness visible light and generate active species that effectively degrade organic pollutants. Ca-AIS exhibits a consistent improvement in visible light photocatalytic performance for 4-NP upon the appropriate doping of Ca^2+^. Nevertheless, it is crucial to exercise caution, as an excessive Ca^2+^ concentration impedes the visible light photocatalytic activity of Ca-AIS toward 4-NP. This suggests that the surplus Ca^2+^ functions as a center for recombination, leading to the recombination of charges and thereby impeding photocatalytic activity.

### 2.6. Photocatalytic Mechanism 

**Figure 6:**
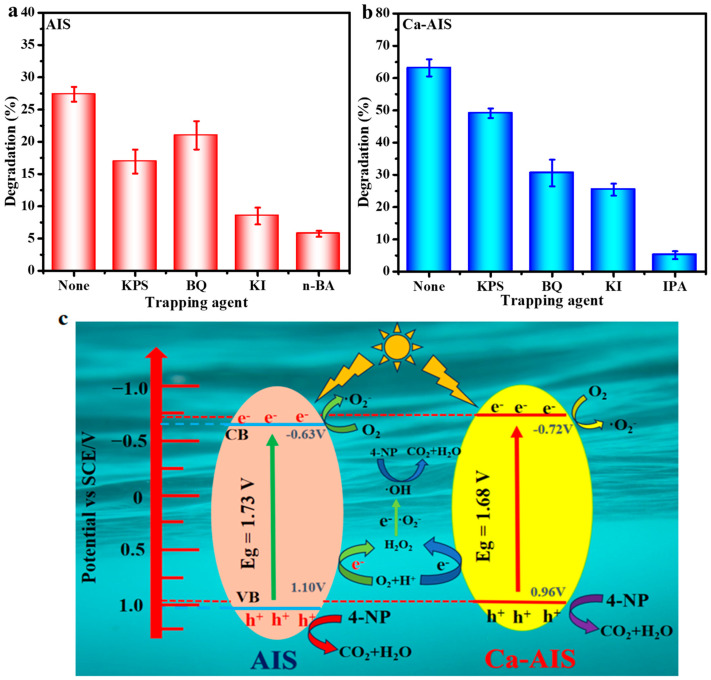
Photodegradation rates of 4-NP by (**a**) AIS and (**b**) 1%Ca-AIS with different active trapping agents and a photocatalysis mechanism diagram under visible light irradiation (**c**).

Generally, during the process of a catalytic reaction, certain active species such as e^−^, ·O_2_^−^, h^+^, and ·OH are produced [47,48]. To determine the active species that play the main role of oxidation and reduction in the catalytic reaction process and thereby speculate the mechanism of the catalytic process, we conducted active species capturing experiments for AIS and 1%Ca-AIS. It is well known that potassium persulfate (KPS), p-benzoquinone (BQ), potassium iodide (KI), n-butanol (N-BA), or isopropanol (IPA) are commonly used as capturing agents for e^−^, ·O_2_^−^, h^+^, and ·OH, respectively [49,50,51]. Figure 6a represents capturing experiments performed over a period of 120 min. Among the species, h^+^ and ·OH play a significant role in the photodegradation process of AIS, followed by e^−^. Figure 6b displays the captured results of active species in 1%Ca-AIS. It is clear that the inclusion of the capturing agent significantly hinders the activity of 1%Ca-AIS in comparison to the photocatalytic degradation of 4- without a capturing agent, indicating that e^−^, ·O_2_^−^, ·OH, and h^+^ all play a role in the degradation of 4-NP by 1%Ca-AIS. The dominant active species in the degradation process of 1%Ca-AIS are ·OH and h^+^, followed by ·O_2_^−^. Based on the analysis, we propose a potential photocatalytic mechanism to elucidate the pathways of charge generation and transfer in Ca-AIS nanomaterials during the photocatalytic degradation of 4-NP. The overview of this mechanism is illustrated in Figure 6c. When AIS and Ca-AIS nanomaterials are exposed to visible light with energy that exceeds the band gap energy, the photon’s energy is absorbed by electrons in the valence band, and they are sent to the conduction band. As a result, electrons (e^−^) are generated in the conduction band, and holes (h^+^) in the valence band are due to the photogenerated process. The production of ·O_2_^−^ can be ascribed to the observation that the CB potential of AIS (−0.63 V vs. SCE) and Ca-AIS (−0.72 V vs. SCE) exhibits a more unfavorable value compared to the redox potential of O_2_/·O_2_^−^ (−0.56 V vs. SCE) [52]. The generation of ·O_2_^−^ occurs when photogenerated electrons are present through a specialized pathway [44]. Since the VB potential of AIS and Ca-AIS is lower than ·OH/H_2_O (OH^−^) (1.75 V vs. SCE), ·OH cannot be generated through the oxidation of H_2_O or OH^−^ by photogenerated holes. Akira Fujishima proposed alternative pathways for the production of ·OH [53]:

(4) $$\begin{array}{l} O_{2}+2e^{-}+2H^{+}\to H_{2}O_{2}0.44\;V(\mathrm{vs}.\;SCE)\\ H_{2}O_{2}+e^{-}\to \cdot OH+OH^{-}0.63\;V(\mathrm{vs}.\;SCE)\\ H_{2}O_{2}+\cdot {O_{2}}^{-}\to \cdot OH+OH^{-}+O_{2}0.76\;V(\mathrm{vs}.\;SCE) \end{array}$$

The VB potential of AIS (1.10 V vs. SCE) and Ca-AIS (0.96 V vs. SCE) are larger than the potential in the reaction formula, so ·OH may be generated through the above pathways in this reaction system. Due to ·OH, h^+^, and ·O_2_^−^ having strong oxidation, they react with 4-NP on the surface of the catalyst to generate CO_2_ and H_2_O and realize the degradation of 4-NP. By employing the inclusion of Ca^2+^, we are able to resolve the instability and compound tendency of h^+^ and e^−^ that are generated by AIS when exposed to light. The inclusion of Ca^2+^ aids in diminishing the resistance encountered during the transfer of electrons at the interface, thereby enhancing the separation and transfer of h^+^ and e^−^. As a result, the recombination of h^+^ and e^−^ is effectively prevented. Moreover, the introduction of Ca^2+^ causes the CB potential of Ca-AIS to undergo a negative shift, resulting in a more reactive and vigilant e^−^. This heightened e^−^ activity participates in the given reaction, actively leading to an increased production of H_2_O_2_, ·OH, and ·O_2_^−^. Furthermore, the consumption of photogenerated electrons frees photogenerated holes for the oxidation reaction, allowing the involvement of more active species in the degradation of 4-NP [54]. Additionally, the narrow band gap of Ca-AIS suggests that it can effectively harness a broader range of light wavelengths, making it suitable for photocatalysis in a wider spectrum of conditions. Such characteristics of Ca-AIS make it a promising candidate for further exploration and development in the field of photocatalysis.

## 3. Materials and Methods

### 3.1. Materials

The experiment utilized analytically pure chemical reagents. AgNO_3_ was procured from Guangdong Guangcai Technology Co., Ltd. (Shenzhen, China)., indium nitrate hydrate was obtained from Shanghai McLean Biochemical Technology Co., Ltd. (Shanghai, China), and thioglycolic acid (TGA) and thioacetamide (TAA) were purchased from Sinopharm Chemical Reagents Co., Ltd. (Beijing, China). Ca(NO_3_)_2_·4H_2_O and isopropanol were acquired from Xilong Science Co., Ltd. (Shantou, China). P-benzoquinone (BQ) and potassium persulfate (KPS) were obtained from Sinopharm Chemical Reagent Co., Ltd. (Beijing, China), and KI was purchased from Tianjin Fengchuan Chemical Reagent Technology Co., Ltd. (Tianjin, China). Ultrapure water with a resistance of 18.25 MΩ cm was utilized in the whole experimental work.

### 3.2. Preparation of AIS and Ca-AIS

AIS: The catalyst was prepared using the low-temperature liquid phase method, with TGA serving as a stabilizer and TAA as the sulfur source [55]. AgNO_3_ (0.2038 g), In(NO_3_)_3_·xH_2_O (1.023 g), and CH_3_CSNH_2_ [TAA (4.5078 g)] solutions were separately prepared in 50 mL of water. A total of 10 mL AgNO_3_, 10 mL In(NO_3_)_3_·xH_2_O, and 0.51 mL C_2_H_4_O_2_S (TGA) were transferred to a round bottom flask and mixed with 370 mL ultrapure water. Subsequently, after intense stirring for 15 min, a 10 mL TAA solution was precisely added to the above mixture under stirring. The mixture was promptly placed in a water bath at a constant temperature of 70 °C. The mixture was allowed to react for 5 h. The reaction system was then removed, cooled, and aged for 24 h to obtain the precipitate of AIS. To ensure the purity of the precipitate, filtration was carried out followed by washing with deionized water until the conductivity of the filtrate matched the deionized water. The desired AIS nanomaterial was obtained by vacuum drying at room temperature for 12 h.

**Figure 7:**
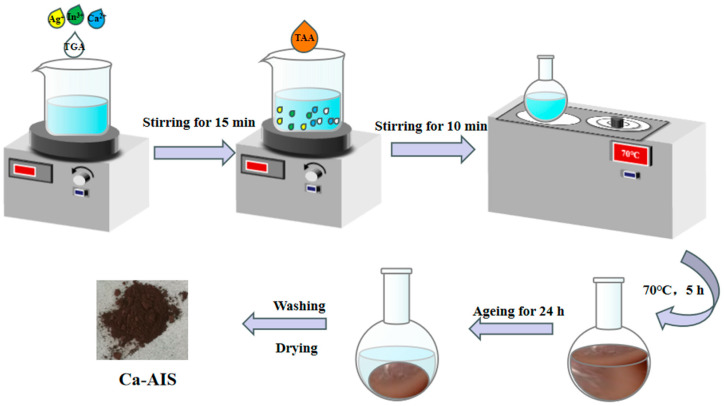
The synthesis roadmap of Ca-AIS.

Ca-AIS: For the preparation of Ca-doped AIS, the same procedure was followed, except Ca(NO_3_)_2_·4H_2_O was added to form a mixed solution of AgNO_3_, In(NO_3_)_3_·xH_2_O and Ca(NO_3_)_2_·4H_2_O with different mass ratios (m (Ca(NO_3_)_2_·4H_2_O):m (AgNO_3_ + In (NO_3_)_3_·xH_2_O) = x%). The addition of Ca(NO_3_)_2_·4H_2_O was just before the addition of TAA to obtain Ca-AIS with mass ratios of 0.5%, 1%, 1.5%, 2%, and 3%. The synthesis roadmap of Ca-AIS is shown in Figure 7.

### 3.3. Characterization

The crystallinity of the samples was analyzed using an X-ray diffractometer. The experiment employed a Cu-Kα as the light source, with the tube voltage set at 40 kV and current at 30 mA. The scanning range spanned from 5° to 90°. Binding energies were measured utilizing an X-ray photoelectron spectrometer, known as Escalab 250Xi (Thermo Fisher Scientific Shier Science & Technology Company, Waltham, MA, USA), and the radiation source employed was Al Ka. This allowed the determination of the type and valence state of the elements. The interface and surface morphology of the samples were examined using a Talos F200X (Thermo Fisher Scientific Shier Science & Technology Company, USA) high-resolution transmission electron microscope (HRTEM), as well as a JSM-7500F (Hitachi, Japan) scanning electron microscope (SEM). To obtain a UV-Vis absorption study of the samples, a TU-1901 UV-Vis (Beijing General Instrument Company, Beijing, China) spectrophotometer was utilized with a measurement range from 200 to 900 nm. BaSO_4_ served as the test background. In our study, we utilized an RF-5301 (Shimadzu, Japan) fluorescence spectrophotometer to acquire the photoluminescence spectrum. The excitation wavelength employed was 400 nm, and the slit widths were set at EX: 6HH and EM: 10. Furthermore, the determination of Brunauer–Emmett–Teller (BET) surface areas was performed using a BSD-660M A3M. Before conducting the analysis, the samples underwent a 2 h degassing procedure at 120 °C.

### 3.4. Photocatalytic Activity

To assess the effectiveness of AIS and Ca-AIS as photocatalysts, a series of experiments were performed utilizing a photocatalytic apparatus. The light source utilized was an iodine tungsten lamp with a power of 1000 W. To eliminate light below 420 nm, a cutoff filter was applied. About 30 mg of the photocatalyst and 80 mL of the 4-NP solution (15 mg/L) were added to a quartz beaker. Before initiating the photocatalytic degradation process, the system was subjected to ultrasonic dispersion for 5 min. Subsequently, it was placed in the photocatalytic reaction device. The distance between the iodine tungsten lamp and the quartz beaker was adjusted to 10 cm. To ensure that the 4-NP diluent attained a state of dark adsorption–desorption equilibrium, the system was stirred in the dark for 30 min. Following this, the light source was turned on to initiate the photocatalytic reaction. The solution was continuously exposed to light for two hours. During this period, 8 mL of solution was taken out after every 20 min. After separation, the optical absorbance of the supernatant was conducted at a wavelength of 317 nm, and the amount of 4-NP degraded was calculated as:

(5) $$D(\%)=\frac{A_{0}-A}{A_{0}}=\frac{C_{0}-C}{C_{0}}\times 100\%$$

In this case, D represents the efficiency of degradation, A_0_ and C_0_ represent the absorbance and concentration of 4-NP prior to being measured after the dark adsorption–desorption equilibrium, and A and C denote the absorbance and concentration of 4-NP during the photocatalytic degradation over a given duration.

### 3.5. Active Species Capture

To investigate the active entities participating in the catalytic process, isopropyl alcohol (IPA) and n-butanol (n-BA) were employed to capture ·OH, while potassium persulfate (KPS), potassium iodide (KI), and p-benzoquinone (BQ) were utilized to capture e^−^, h^+^, and ·O_2_^−^ separately. The same procedure was followed, as described for the measurement of the photocatalytic activities, except a small amount of the capturing agent was added in each case.

### 3.6. Flat Band Potential Test

To conduct the test for the flat charge potential of the catalyst, an electrochemical method with a three-electrode setup was employed. The experimental arrangement comprised three electrodes, namely the working electrode, reference electrode, and counter electrode. The working electrode consisted of a conductive glass of indium tin oxide (ITO) coated with a catalyst, the reference electrode was a saturated calomel electrode (SCE), and the counter electrode comprised platinum in a 0.1 mol/L Na_2_SO_4_ solution. The ITO glass (measuring 1 cm × 2 cm) was cleaned completely using distilled water, acetone, and ethanol. The first step in the preparation of the working electrode involved dispersing a 30 mg catalyst in 10 mL of ethanol. The mixture was ultrasonically treated for 30 min. Subsequently, 10 mL of ethylene glycol was added, followed by another 30 min ultrasonic treatment. The resulting mixture was magnetically stirred for 5 h, resulting in a viscous dispersion containing the catalyst. A droplet of the dispersion measuring 20 μL was then carefully placed on the ITO conductive glass. The glass was left undisturbed for 1 h and was subsequently dried using an infrared lamp to obtain the working electrode. To remove any traces of O_2_ in the electrolyte, the solution underwent N_2_ bubbling for 30 min. We conducted an analysis of the voltage stability using the “open circuit potential-time” method. A scanning voltage range of −1 V to 1 V we selected utilizing the “impedance-potential” method. The flat band potentials of the samples were determined by analyzing the information obtained from this curve to measure the Mott–Schottky (M-S) curve.

### 3.7. Photoelectric Chemical Test

Photocurrent tests were used to detect the response strength of the catalyst to light and the carrier separation efficiency. We utilized the CHI-66D model of the electrochemical workstation to assess the homeopathic photocurrent response (I-t) of the functioning electrode. Moreover, we designated the initial voltage as the stable voltage of the open circuit potential. Electrochemical impedance spectroscopy was used to detect the charge transfer rate. In this work, the electrochemical impedance model was CHI-66D, the test amplitude was 5 mV, and the frequency was 1–10^5^ Hz under open circuit voltage.

## 4. Conclusions

The main objective of this investigation was to fabricate pristine AIS and Ca^2+^-doped AIS utilizing the liquid phase method at low temperatures. The structural arrangement of AIS crystals remained unaltered, even after the addition of Ca^2+^. The detectable photocatalytic performance of Ca-AIS exhibited an escalation within a specific scope as the concentration of Ca^2+^ dopants increased. When the Ca^2+^ concentration was maintained at 1%, Ca-AIS was subjected to visible light for a duration of 120 min, and the degradation efficiency of P-Nitrophenol reached an impressive 63.2%. Despite the potential benefits of incorporating Ca^2+^ for improving the visible photocatalytic activity of AIS, it was observed that excessive Ca^2+^ content had a detrimental effect on the activity. Furthermore, Ca-AIS exhibited superior sphericity, smaller particle size, more uniformity in size, better dispersibility, and more active sites. Capturing experiments confirmed that the active species engaged in the degradation of 4-NP were ·OH and h^+^. The introduction of Ca effectively suppressed the recombination of h^+^ and e^−^ and generated more ·O_2_^−^, resulting in improved photocatalytic activity of AIS. The target pollutant in this work was organic pollutant (4-NP) wastewater simulated under laboratory conditions. The complex actual wastewater situation in which multiple pollutants coexist has not been further explored. Therefore, it is necessary to explore the application of this system to composite pollutants and actual wastewater. Follow-up research can broaden its application areas to photocatalytic water splitting for hydrogen production and even try to treat some real industrial wastewater.

Furthermore, Ca-AIS can also be doped with different extraneous elements in an attempt to modify its energy band structure and electronic state, and then it can be compounded with magnetic semiconductors to find better combinations for achieving higher photocatalytic performance. Subsequently, these optimized combinations can be used in photocatalytic hydrogen production or acoustic catalysis experiments.

## Data Availability

Data are contained within the article.
