# Correction: Wang et al. Visible Light Motivated the Photocatalytic Degradation of P-Nitrophenol by Ca^2+^-Doped AgInS_2_. *Molecules* 2024, *29*, 361

**DOI:** 10.3390/molecules29122764

**Published:** 2024-06-11

**Authors:** Xuejiao Wang, Shuyuan Liu, Shu Lin, Kezhen Qi, Ya Yan, Yuhua Ma

**Affiliations:** 1 College of Pharmacy, Dali University, Dali 671000, China; 15187277683@163.com (X.W.);; 2 College of Chemistry and Chemical Engineering, Xinjiang Normal University, Urumqi 830054, China

In the original publication [1], there was a mistake in the order of existing authors. The first author should be Xuejiao Wang.

The correct authorship of this paper should be:

Xuejiao Wang ^1^, Shuyuan Liu ^1^, Shu Lin ^1^, Kezhen Qi ^1,^*, Ya Yan ^1,^*, and Yuhua Ma ^2,^*

The authors state that the scientific conclusions are unaffected. This correction was approved by the Academic Editor. The original publication has also been updated.
